# Gender Perspectives on Social Norms Surrounding Teen Pregnancy: A Thematic Analysis of Social Media Data

**DOI:** 10.2196/13936

**Published:** 2019-09-17

**Authors:** Kathryn M Barker, S V Subramanian, Robert Selman, S Bryn Austin

**Affiliations:** 1 Center on Gender Equity and Health University of California San Diego La Jolla, CA United States; 2 Harvard TH Chan School of Public Health Boston, MA United States; 3 Harvard Graduate School of Education Cambridge, MA United States

**Keywords:** teenage childbearing, teen pregnancy, adolescent sexual behavior, social media, social norms, gender

## Abstract

**Background:**

Social concern with teen pregnancy emerged in the 1970s, and today’s popular and professional health literature continues to draw on social norms that view teen pregnancy as a problem—for the teen mother, her baby, and society. It is unclear, however, how adolescents directly affected by teen pregnancy draw upon social norms against teen pregnancy in their own lives, whether the norms operate differently for girls and boys, and how these social norms affect pregnant or parenting adolescents.

**Objective:**

This research aims to examine whether and how US adolescents use, interpret, and experience social norms against teen pregnancy.

**Methods:**

Online ethnographic methods were used for the analysis of peer-to-peer exchanges from an online social network site designed for adolescents. Data were collected between March 2010 and February 2015 (n=1662). Thematic analysis was conducted using NVivo software.

**Results:**

American adolescents in this online platform draw on dominant social norms against teen pregnancy to provide rationales for why pregnancy in adolescence is wrong or should be avoided. Rationales range from potential socioeconomic harms to life-course rationales that view adolescence as a special, carefree period in life. Despite joint contributions from males and females to a pregnancy, it is primarily females who report pregnancy-related concerns, including experiences of bullying, social isolation, and fear.

**Conclusions:**

Peer exchange in this online forum indicates that American adolescents reproduce prevailing US social norms of viewing teen pregnancy as a social problem. These norms intersect with the norms of age, gender, and female sexuality. Female adolescents who transgress these norms experience bullying, shame, and stigma. Health professionals must ensure that strategies designed to prevent unintended adolescent pregnancy do not simultaneously create hardship and stigma in the lives of young women who are pregnant and parent their children.

## Introduction

Teen birthrates in the United States have been steadily declining since the mid-1990s [1]. In 2017, a total of 194,377 babies were born to American women aged 15-19 years, for a live birth rate of 18.8 per 1000 women (or approximately 2% of women) in this age range [2]. In the United States and other industrialized societies today, teen childbearing is viewed as a concern because adverse health and social outcomes have been observed among teen mothers and their children, including infant mortality, childhood illness, welfare dependence, academic failure, juvenile crime, and teen childbearing in subsequent generations [3,4]. A contentious debate exists in the academic literature regarding whether these negative associations reflect preexisting differences between teens who gave birth compared to those who did not [5,6]. For example, studies adjusting for background characteristics associated with teen pregnancy (eg, family background, race/ethnicity, socioeconomic position, and educational attainment) show that teen mothers are as likely as older mothers to bear and raise healthy, successful children [7-12] and that life trajectories of teen mothers are slightly altered by having children in their teens [7,13,14].

**Figure 1:**
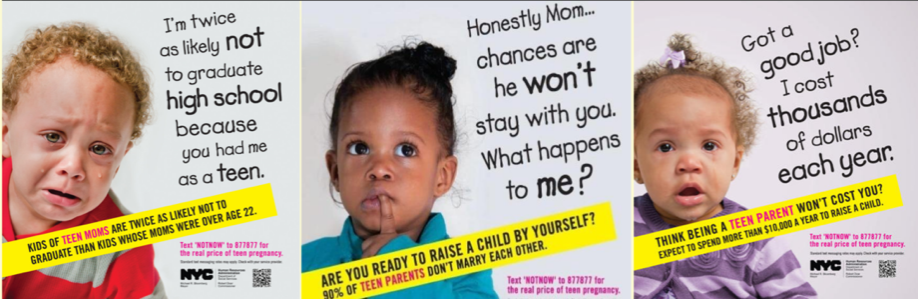
NYC 2013 Teen Pregnancy Prevention campaign materials.

Despite this evidence, which calls into question the validity of causal assumptions between teen parenthood and poor social and economic outcomes and the fact that current teen birthrates are the lowest rates ever recorded in the United States [2], national concern with teen pregnancy remains [15]. A steady stream of professional and popular literature continues to circulate the narrative that early childbearing leads to considerable and devastating costs to the public and teen mothers and their children (for example, materials from New York City’s controversial 2013 campaign against teen pregnancy; Figure 1 [16]). Indeed, the dominant narrative in the United States deems teen pregnancy a social and public health problem [17-21], unequivocally bad for young women, their children, and society [22]. This dominant narrative is represented and mutually reinforced in multiple arenas including academic literature, media and popular discourse, and social policy [22,23].

The social ramifications of adolescent fertility vary by time and context, with US social concern with the concept of “teen pregnancy” dating roughly to the 1970s [3,20,24]. This concern is informed by a shifting set of social norms in American society [25]. Social norms are group-level expectations for appropriate behavior that result in negative sanctions such as feelings of embarrassment, anxiety, guilt, and shame for individuals who violate them [26-29]. Social norms regulating pregnancy are informed by societal views on the acceptability of teen sex, contraception, pregnancy, and abortion [25] as well as age and gender norms. Childbearing is typically not discouraged when it occurs in adulthood and after transitioning to marriage and financial independence [30]. Although norms against sex and pregnancy during adolescence apply to both males and females, normative standards are generally enforced more strongly for girls than boys [31]. Despite the importance of gender in understanding teen pregnancy norms, much of the research on teen pregnancy investigates the roles of race and class and is relatively silent with respect to gender [32].

American adolescents increasingly access online communities for social support and advice about personal matters [33-37]. These digital platforms allow adolescents opportunities to explore their identity and find support and information about developmentally sensitive issues, such as sexual health [38]. In a study of online health-seeking behaviors, it was found that 44% of individuals aged 15-24 year who have looked for health information online sought out information about sexual health, second only to information about diseases such as cancer [39]. This is, perhaps, unsurprising given that youth often feel uncomfortable directly asking parents, teachers, and physicians about topics such as pregnancy, sexuality, menstruation, and sexually transmitted infections [40,41]. This makes digital platforms such as online social networks a highly relevant data source for examining adolescents’ views of teen pregnancy. Use of these types of online data is in line with existing qualitative methodologies that adapt traditional ethnographic techniques to the study of social media [42,43].

The online ethnographic research method used here draws on adolescents’ anonymous, voluntary, and intentional interactions with other online users in the exchange of information and advice about teen pregnancy in a popular social media site to answer the following research questions: Are the views of contemporary adolescents shaped by norms against adolescent pregnancy? If yes, how do US adolescents utilize, reproduce, or reinterpret these dominant narratives in their discussions of teenage pregnancy? In addition, how do these norms shape the lives of contemporary teens who experience pregnancy? Given the declining rates of teen pregnancy in the United States and the variation in pregnancy norms by time and context, this research allows for the examination of whether and how the people affected by these social norms—US adolescents—use, interpret, and experience social norms against teen pregnancy.

## Methods

### MTV’s “Over the Line?” Platform

In 2009, MTV launched “Over the Line?” an online social networking site for youth aged 14-24 years to give and receive feedback and support on life experiences [44]. This popular site has over 18,000 followers [45]. Although MTV designed the platform with the intention of providing space for adolescents to share experiences of digital abuse, adolescents ultimately used the platform for multiple topics including sexual and reproductive health concerns.

As shown in Figure 2, users could interact on this platform in multiple ways. First, users seeking advice could post a comment describing their concern along with the option to provide their name, age, or gender. Second, for each user comment, other users could vote on whether the comment was “over,” “on,” or “under” the line. These ratings were used to indicate the acceptability of the behavior described in the comment, with “over-the-line” ratings indicating an unacceptable action or behavior. Third, online users could also provide written responses to each comment. Thus, there are three different types of data available for analysis on this platform: the original comment, quantitative votes on the acceptability of the issue presented in the comment, and qualitative responses to the comments.

These data represent a novel approach to gaining insights into the concerns expressed by adolescents surrounding pregnancy and pregnancy-related issues. Traditional qualitative methods (eg, in-depth interviews) necessarily involve a relationship between the researcher and the participant [46], which mobilizes and may reproduce the structural positions that exist outside research contexts and thereby risk perpetuating existing power relationships [47]. This is of concern because teens have a general reluctance to discuss health and sexuality concerns with parents and health providers due to embarrassment and concerns about privacy [48,49]. The anonymous peer-to-peer exchange available to teens in MTV’s Over the Line platform eschews these existing concerns and methodological issues. In addition, the MTV platform was open and available to any adolescent with access to the internet. Using this population of teenagers in online platforms allows for expansion beyond traditional contextual or localized studies of teen pregnancy. The data corpus for this analysis comprises three different types of data posted to MTV’s Over the Line platform between March 2010 and February 2015: (1) pregnancy-related *comments* made by individuals between the ages of 13 and 19 years (n=208) and the subsequent (2) quantitative (n=15,445) and (3) qualitative *responses* (n=1454) associated with each comment.

**Figure:**
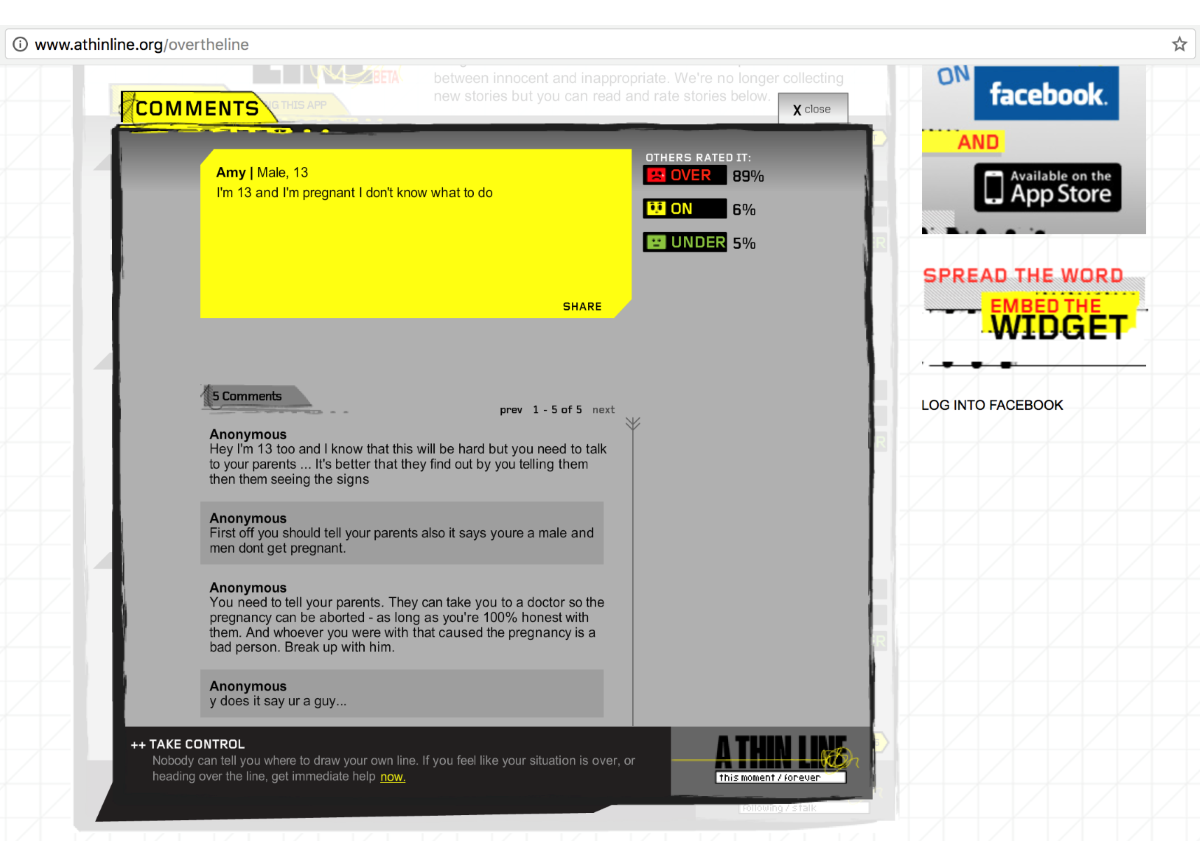
Exemplar “Over the Line?” comment and associated quantitative and qualitative responses.

### Analysis

Qualitative analysis progressed in several steps. The first step used thematic analysis to identify and isolate comments related specifically to adolescent pregnancy from the entire corpus of the MTV OTL platform. Once this subsample of all pregnancy-related comments was identified, emic codes were developed using thematic analysis [50,51]. The first author read through all comments to become familiar with the data and identify preliminary themes. Subsequently, a 10% random sample of the full sample of 208 comments was selected to develop inductive or emic codes. Following initial code identification, analytical themes were identified and conceptualizations of each theme were refined.

Throughout this process, a codebook was developed and refined, as informed by both the first author’s own interpretations of the data after closely reading through the data as well as a multistep, interrater reliability (IRR) process. After the initial codebook development, the first author and a coder trained in thematic analysis coded a new random sample of 20 comments. The two researchers then held a consensus interrater discussion to clarify areas of disagreement and identify codes requiring refinement. The codebook was then revised, and a second IRR test was performed using another random sample of 30 stories. A coding-comparison query was conducted in NVivo 10 software (Doncaster, Australia). Kappa statistics were calculated for each code and high levels of agreement (ie, κ>0.75 [52,53]) were achieved after two rounds of reliability testing.

One critical emic code, “Value Statements or Judgments Surrounding Teen Pregnancy,” was identified in this process. After consultation with other authors, the first author sought to develop related etic codes from extant conceptual frameworks in qualitative studies on normative contexts of early childbearing. Three such studies were identified [22,31,54], and 10 resulting etic codes on social norms were used in analysis. Subsequently, a second codebook on social norms was developed specifically for the analysis of comments and associated responses that were initially coded as “Value Statements or Judgement Surrounding Teen Pregnancy.” In the final phase of analysis, the first author checked the integrity of the themes in relation to their component coded extracts as well as to the dataset as a whole.

## Results

### Female Perspectives

Of the 208 total comments about teen pregnancy, the vast majority (n=195, 94%) were posted by females, and the subsequent analysis is restricted to comments made by females. These comments centered on two major themes: gauging the social acceptability of wanting to have a child in their teenage years and sharing experiences with bullying, social isolation, and fear due to actual or rumored pregnancy. Given the large body of data and the research focus on norms, the results are structured as follows: first, comments related to the social acceptability of wanting a baby as a teenager are examined; second, the responses to these comments are analyzed; finally, female users’ experiences with bullying, social isolation, and fear due to pregnancy are presented.

### Comments: Gauging the Social Acceptability of Teen Pregnancy and Motherhood

A number of adolescents (all but one female) used the Over the Line platform to gauge the social acceptability of wanting to have a child in their teenage years. These advice-seeking comments indicated the commenters’ recognition of age norms regulating the timing of pregnancy. For example, “I want to had a baby but I’m 13. Is that wrong?” Other uses were more explicit in their awareness of age norms and pregnancy timing:

Im sixteen and want a baby more then anything, i know im young and with only being a junior in high school i understand it would affect things & still i want one, what should i do?

A minority of commenters provided a rationale for why they wanted to have a child, which were related to important life transitions such as marriage:

I'm 16 almost 17. I'm engaged getting married next summer. I really wanna have my fiances baby now.

As opposed to other comments that centered on experiences of pregnancy and bullying, these advice-seeking comments elicited the vast majority of responses from other online users. Given that the advice one provides is reflective of the beliefs one holds [55], the analysis of responses to these comments provides insights into the normative contexts in operation in this online platform.

### Quantitative Votes on the Acceptability of Adolescent Childbearing

A total of 4292 Over the Line platform users provided quantitative responses on whether comments related to wanting a child as a teenager were over the line: 88% of platform users voted for over the line (ie, unacceptable); 7%, for on the line; and 5%, for under the line (ie, not a problem). When disaggregated by original commenters’ age, votes indicated stricter age norms for younger adolescents; 90% of voters felt it was unacceptable for adolescents aged 13-15 years to consider having a baby, as compared to 81% for those aged 16-19 years. In general, a clear majority of Over the Line platform users felt teen pregnancy at *any* age was unacceptable. Users responded with many reasons for why it was unacceptable.

### Qualitative Responses About Adolescent Childbearing

As with the quantitative data, qualitative responses overwhelmingly reflected the dominant view in the United States that teen pregnancy is problematic. This view was reflected through several subnorms (shown in italics). Some of the user comments reflected a general *pathologization* of teen pregnancy without any specific mention of *why* it was problematic. The lack of a need for explanation is indicative of how strong this norm against teen pregnancy is for some individuals. For example, a user responding to a teen wanting a baby simply said, “dont be stupid about this crap okay make a smart choice.” Most user responses did, however, include rationales to explain why having a baby as an adolescent was a bad idea. Age norms informed many rationales. In some cases, young age was the sole rationale for why becoming pregnant was a bad idea. For example, one user noted, “You shouldn’t even be having sex at 13?!!” *Missed adolescence* reflects a view that adolescence should be a fun and carefree time, unburdened by responsibilities associated with parenthood. Users commented as follows:

Enjoy your childhood, spend time with your bf, go on road trips! You cant do any of that easily with a baby

Enjoy teenhood with partys not diapers.

Many users focused on the level of responsibility to dissuade fellow peers from having a baby. The theme, *Too much responsibility*, explicitly indicated concerns with the amount of time and effort required to care for a baby. As one respondent stated, “yhuu may thiink yhuu wnt t2 but no yhuu not ghunna really wnt it wen yhu have t2 ghett upp in dha mornin t2 b changing diapers and hearin ya bby yelling [you may think you want to have a baby, but you’re not going to really want it when you have to get up in the morning to change diapers and hear your baby yelling].” Many commenters drew on age norms when describing the level of responsibility involved in having a baby:

A baby is a HUGE responsibility and what 13 year old likes any responsibility of any kind?

Other commenters drew upon the *Children having children* narrative:

You have NO idea how to raise a child when ur still one urself...wud u be ready for staying up throughtout the nite, constant crying n buying everything.

This theme implicitly informed another theme: *Limited future opportunities.* Here, user responses ranged from beliefs indicating a baby would delay or limit the attainment of life goals (for example, “Having a kid will delay your education and your childhood”) to those totally derailing chances at a good life (for example, “The chances of you being poor, unstable and unhappy for the rest of our life are very high having a baby that young and unmarried.”) Other users were not as concerned about the future of the adolescent, but the future of the baby. Overarching warnings within the theme *Not good for baby* theme included, “the baby wouldn’t be happy.” More specific warnings ranged from predictions that the child would receive limited education or would eventually also become a teen mom:

If you have a baby now there is a huge chance that that child wont finish its education and will also be a prego teen.

This theme had overlapped with *Instability* to some extent, wherein user responses focused on the unstable nature of adolescents’ relationships and finances. Concerns of relationship instability related to a sentiment that intense feelings for romantic partners during adolescence are fleeting:

Its called puppie love - 6 months from now ull be in “love” with some1 else.

Other users warned that adolescent males just wanted sex, and a boyfriend would likely leave if the commenter became pregnant:

Do not trust boys you know all they want is sex.

Ya man gone end upp leaving yhuu [you] cuz he gone thiink yhuu [you’re] t2 [too] much.

User responses also focused on adolescents’ limited independence and the continued reliance on parents:

If you want a baby at 14 you are obviously not thinking, you cant finacially support the baby your parents would have to.

Interestingly, in this sample, the concern with the impact on others focused on familial burden and not societal burden. Only one response in the entire dataset drew on the *Welfare queen* trope to explain why teen pregnancy was a problem. In addition, a very small minority of user responses centered on *Moral rationales* to explain why sexuality was problematic at this age:

Most of these children these days need to read their bible.. Your not suppose to have sex until your married.

Finally, other user responses centered on *Pathologized (female) adolescent sexuality*, illustrating that for some users, the problem is not with teen pregnancy or the challenges of raising a baby as an adolescent, but with sexuality:

***^^^OKAY EVERYONE IS SAYING YOUR TOO YOUNG TO HAVE A BABY; HOW ABOUT YOUR TOOO YOUNG TO HAVE A BOYFRIEND AND YOUR TOO YOUNG TO BE HAVING SEX.

Another user response illustrated that this concern centers on the sexuality of girls, drawing on age, gender, and sexual norms, for example, the following response, which is a gendered statement that aims to chasten female sexual desires:

Keep your **** legs close you are to young.

None of the pregnancy comments in this dataset implicated male partners as being sexually irresponsible. This reflects dominant US societal norms that deem adolescent female—not male—sexuality as problematic [20].

### Female Experiences of Bullying, Social Exclusion, and Fear

About half of the commenters sought advice about negative experiences related to bullying, social isolation, and fear due to an actual or rumored pregnancy, and all but two of these comments were shared by female users. Female adolescents shared stories about being bullied because they had been pregnant or by being told they were or looked like they were pregnant. In these stories, teen pregnancy was mentioned in context of girls being “sluts” or “whores,” which are derogatory terms used to disparage women for having many sexual partners [56]. As one respondent noted:

I would never want a kid at this age…people will call you a slut.

This very scenario indeed played out in the lives of some adolescent girls:

People im school call me a **** becasue i had a baby when i was 17 i dont know if i should jus ignore it and think that im better then that or do something.

People started these nasty rumors about me being prego and aborting the baby, and ingraved “slut” on my locker.

As an indication of the extent of the damaging nature of this bullying, one poster shared that she chose to leave school to escape rumors that she was pregnant:

This girl at my school told everyone hat i was having sex with my boyfriend. but i wasn't. I couldn't deal with it so i broke up with him and tried 2 keep 2 myself. She started telling people i was pregnant. I left school and moved 2 a different 1.

Many of the female commenters also experienced social isolation and fear after a pregnancy. These types of comments indicate that the responsibility for the pregnancy rested with the girl, as opposed to a shared responsibility by both sexual partners. One young woman wrote:

Im 17 yrs old and met this guy about 4 months ago and we had sex once. i got pregnant and now he wants nothing to do with me or the baby!!

Another user was worried about the repercussions of telling her family:

I found out i was pregnant & i'm scared to tell my parents.

In the worst-case scenarios, norms of teen pregnancy as a social problem and “slut shaming” led to both bullying and social isolation in the lives of young women, as illustrated by the following comment:

My bf took naked pics of me and video taped us having sex and all of my friends left me and I dumped him but now he’s saying that I’m a ***** and I’m pregnant with this kid and he’s saying it isn’t him. Please help me!!!!! The whole school knows!!!!!

## Discussion

### Principal Findings

This study draws on a novel methodology to reveal a number of key insights into how American adolescents in a popular online forum reproduce, reinterpret, and are affected by dominant American social norms surrounding teen pregnancy. First, users seeking to gauge the social acceptability of wanting a baby in their teen years received responses that were largely reflective of norms pathologizing teen pregnancy. A majority of the online respondents viewed any type of teen pregnancy to be problematic, with 88% of users indicating that comments related to having a child as a teenager were unacceptable. The vast majority of qualitative responses reflected subnorms against teen pregnancy. Second, these data highlight important ways in which pregnancy norms are also informed by age, gender, and sexual norms. On disaggregating the data according to age, there was a stronger disapproval for pregnancy in younger teens (ie, those aged 15 years or younger) as compared to older teens. This is in line with previous research indicating that teenage mothers below the age of 15 years tend to arouse more concern than their older counterparts [57]. Users also drew upon gender and sexual norms to provide rationales against teen pregnancy. The data highlight the gendered concerns of young women as they negotiate their sexuality in the context of social norms that pathologize teen pregnancy. Finally, these data indicate that norms against teen pregnancy create a social environment that may lead to stigma, shame, and difficulty in the lives of young women who experience a pregnancy. Whether the absence of such stories in this platform among males is a reflection of their limited experience with such stigma or a lack of desire to share such stories in online settings is unclear.

### Future Research

Sociological work by Vinson [58] points to the fact that men are largely absent in the dominant narrative of teen pregnancy, and the social anxiety about teen pregnancy largely focuses on women’s bodies, desires, and morality. Previous quantitative research on social norms assessing adolescents’ perceptions of teenage pregnancy shows clear gender differences: Girls were significantly more likely to report embarrassment at the prospect of a teenage pregnancy than boys [59]. In addition, prior qualitative research on teen pregnancy indicated that social concern among school-aged respondents centered on the female but not the male partner [31]. The public health literature is surprisingly, and perhaps, concerningly silent in its explicit mention of gender and the roles that young men play in contributing to pregnancy. Additional research must be conducted to determine how these norms may play out in the lives of US teenage males.

Second, there is limited clarity in social norms surrounding teen parenthood versus teen pregnancy. Formative research for this study demonstrated that a distinction is only sometimes made between teen pregnancy and teen parenthood in the literature [60], and it is not always a careful one. This is unfortunate, as the constructs of teen pregnancy and teen parenthood are arguably quite distinct and have gendered elements. There is ample room for additional research to disentangle these two constructs and examine how gender norms are utilized in discourse on teen parenthood versus teen pregnancy.

Finally, the dominant social narratives on adolescent childbearing in the United States are based on both race and class [22], and disparities in US teen birth rates are reported by ethnicity and socioeconomic status [59,61,62]. Data limitations here did not, however, permit an exploration of normative differences by demographic characteristics beyond age. Additional data are needed to enable a careful comparative analysis of how social norms against teen pregnancy operate across racial and class lines.

### Conclusions

This analysis provides novel and valuable contributions to the literature by filling a gap in our understanding of teen pregnancy norms. Using a gender lens, the analysis provides a nuanced view of how US adolescents in online platforms use norms surrounding teen pregnancy, and demonstrates how these norms negatively affect the lives of young women who are rumored to be or are pregnant. Our study indicates that teen pregnancy norms disproportionately affect young women and operate to create stigma and social isolation in the lives of adolescent girls. The fact that this population faces stigma is a concern from not only a reproductive justice standpoint, but also a public health standpoint; perceptions of judgmental attitudes lead some pregnant female adolescents to delay accessing health services to avoid judgement [23].

The norms against teen pregnancy remain in place, in part, due to a belief that rejecting the stigmatizing norms against teen pregnancy would be seen as synonymous with encouraging (young women’s) irresponsible and risky sexual behavior [58]. This is a false binary. Demanding more respect for young pregnant and parenting women and rejecting these stigmatizing norms can and should leave room for efforts to prevent unintended pregnancy by improving access to low- and no-cost contraception and comprehensive sexual and reproductive health programs. This is in line with numerous efforts rooted in the principles of reproductive justice [63,64]. By rejecting norms that center on gendered and classed ideologies of motherhood and those that pathologize young women’s, but not young men’s, sexuality, we foster the required social discourse on the structural drivers of unintended pregnancy and the contributing role of young men in pregnancy.

In a social policy book on US teenage childbearing written over two decades ago, sociologist Kristin Luker asked, “How can society’s concern about teenagers and their babies be mobilized to good effect? How can such anxiety be made less confused and inchoate—be made to reflect real problems? Most centrally, how can society ensure that this anxiety—which relates to sexuality, race, poverty, gender, and a changing world economy—not simply exacerbate the existing problems of young women and their babies?” [57]. These questions remain salient but unanswered. American young women and their children would be well served by having researchers, health professionals, and social policy analysts finally answer these difficult but necessary inquiries. It is crucial for public health professionals to ensure that campaigns designed to prevent unintended adolescent pregnancy do not simultaneously create unnecessary hardship for pregnant and parenting young women.

## Abbreviations

IRR: interrater reliability
